# Risk Factors of COVID-19 Critical Outcomes in the Eastern Mediterranean Region: Multicountry Retrospective Study

**DOI:** 10.2196/32831

**Published:** 2022-03-15

**Authors:** Faris Lami, Maisa Elfadul, Hiba Rashak, Mohannad Al Nsour, Hashaam Akhtar, Yousef Khader, Ahmed M Hussein, Mariam Naciri, Sahar Samy, Yasser Ghaleb, Hana Taha, Alaa Hussein, Nameer A Ali, Raheem Hussein, Aamer Ikram, Fazal ur Rahman, Mohammad Mujeeb Khan, Reema Adam, Abdulrazaq Yusuf Ahmed, Salma Afifi

**Affiliations:** 1 Department of Community and Family Medicine University of Baghdad Baghdad Iraq; 2 Public and Tropical Health Programs Epidemiology and Biostatistics University of Medical Sciences and Technology Khartoum Sudan; 3 Ministry of Health Directorate of Public Health Baghdad Iraq; 4 The Eastern Mediterranean Public Health Network Global Health Development Amman Jordan; 5 Yusra Medical and Dental Collage Yusra Institute of Pharmaceutical Sciences Islamabad Pakistan; 6 Faculty of Medicine Department of Community Medicine, Public Health and Family Medicine Jordan University of Science and Technology Amman Jordan; 7 Public Health Department Somali International University Magadishu Somalia; 8 Research Center Biotechnology, Biodiversity and Environment, Laboratory of Biodiversity, Ecology and Genome, Biology Department Faculty of Sciences Mohammed V University Rabat Morocco; 9 Communicable Disease Control Department - Preventive Sector Ministry of Health and Population Cairo Egypt; 10 Yemen Field Epidemiology Training Program Ministry of Public Health and Population Sana’a Yemen; 11 Department of Basic Medical Sciences Faculty of Medicine The Hashemite University Zarqa Jordan; 12 Public Health Department Ministry of Health Babel Health Directorate Babel Iraq; 13 Public Health Department Ministry of Health Al-Rusafa Health Directorate Baghdad Iraq; 14 National Institute of Health Islamabad Pakistan; 15 Medical Unit 1 Benazir Bhutto Hospital Rawalpindi Medical University Rawalpindi Pakistan; 16 Infectious Diseases Department Rawalpindi Medical University Rawalpindi Pakistan; 17 Federal Ministry of Health Directorate of Emergency and Humantarian Actions Khartoum Sudan; 18 Minstry of Health Demartino Hospital Mogadishu Somalia; 19 Ministry of Health and Population Cairo Egypt

**Keywords:** critical outcomes, COVID-19, severity, mortality, outcome, risk factor, retrospective, implementation, demographic, pattern, trend, risk

## Abstract

**Background:**

The establishment of empirical evidence in the Eastern Mediterranean Region necessitates the implementation of wide-scale studies to describe the demographic, clinical features, and severity profile of patients with COVID-19.

**Objective:**

This study aims to assess the patterns of COVID-19 severity and mortality in seven countries, and to determine the risk factors of COVID-19 severity and mortality.

**Methods:**

This multicountry study was based on a retrospective review of medical records of hospitalized patients confirmed to have COVID-19. This study includes data from Iraq, Pakistan, Sudan, Somalia, Morocco, Egypt, and Yemen. All demographic and clinical data were extracted from hospital records (paper files) by trained data collectors.

**Results:**

A total of 4141 patients were included in this study from seven countries. Comorbidities were reported by nearly half of the patients, with hypertension (n=1021, 24.7%) and diabetes (n=939, 22.7%) being the most common. Older age, diabetes mellitus, hypertension, and heart diseases were significantly associated with COVID-19 severity and mortality. Ever smoking and renal diseases were significantly associated with severity but not mortality, while male gender, respiratory diseases, and malignancy were significantly associated with mortality but not severity.

**Conclusions:**

The study confirms the role of comorbidities and demographic features on the severity and mortality of COVID-19. Understanding the contributing factors ensures attentive care and informs clinical management of patients with poorer prognoses in the early stages of diseases.

## Introduction

On December 31, 2019, cases of pneumonia of unknown etiology were reported from Wuhan City, Hubei Province of China [1]. Later, SARS-CoV-2 was identified as a causative agent, and the disease was named COVID-19. The World Health Organization (WHO) declared SARS-CoV-2 to be a global pandemic on March 11, 2020 [1,2]. Globally, 200,840,180 cases of COVID-19 were confirmed and 4,265,903 deaths were reported as of August 6, 2021. In the Eastern Mediterranean Region (EMR), a total of 12,949,856 cases and 240,395 deaths were reported as of August 6, 2021 [3]. The recent forecasting by Institute for Health Metrics and Evaluation has projected COVID-19–related deaths in North Africa and the Middle East to reach up to 327,956 deaths by December 2021 [4].

As many of the countries in the region are experiencing war and political instability, cases and deaths are underreported because of inadequate testing facilities, weak health system response, and inadequate vital registration and documentation [5]. The high hospital admissions and poor surge capacity of critical care were experienced as one of the key health facility challenges that would undermine the response to the COVID-19 pandemic [5]. Difficulties in the estimation of COVID-19 hospital admission rates were highlighted early during the pandemic, as it depends on community testing and admission criteria, which varies between countries. Studies from China, Europe, and the United States have indicated rates of admission to intensive care ranging from 5% to 32%, respectively [6-9].

A recent report showed that Iran has accumulated the highest number of cumulative deaths (n=85,694, case-fatality rate [CFR] 2.6%), followed by Pakistan (n=22,582, CFR 2.3%) and Iraq (n=17,515, CFR 1.2%) [10]. Whereas, Yemen reported the highest CFR (19.7%) followed by Sudan (7.5%). The lowest CFR was reported from Qatar (0.2%) [10]. The difference in the population profile and health system capacity between countries might result in variation in epidemiological characteristics and clinical outcomes. It is worth noting that the Middle East region has a high prevalence of diabetes and cardiovascular diseases (CVDs) that may contribute to heighten the severity of the disease resulting in high mortality and lifelong disability [11-13].

Severe cases of COVID-19 are more likely to deteriorate to conditions that necessitate vital and timely care [6,7]. Epidemiological studies in China, Canada, the United States, and the United Kingdom have identified risk factors associated with severe cases of COVID-19 that require hospital admission [6-9]. Old age, chronic comorbidities, and male sex have consistently been cited as risk factors associated with COVID-19 severity and increased mortality [14-16]. Other risk factors including chronic obstructive pulmonary disease (COPD) and smoking were reported in other studies [17].

Despite the fact that there is limited evidence on the severity of COVID-19 across the EMR, recent studies from Iraq were consistent with global evidence that old age, male gender, and pre-existing comorbidities were associated with increased mortality among hospitalized patients with COVID-19 [18,19].

The establishment of empirical evidence in the EMR necessitates the implementation of wide-scale studies to describe the demographics, clinical features, and severity profiles of patients with COVID-19. Assessing the risk factors of the severe form of COVID-19 is essential to develop appropriate risk reduction strategies and to plan resources for health care as the pandemic unfolds. Furthermore, it will inform clinical management by predicting clinical outcomes and prognostic markers to facilitate the development of a care pathway for COVID-19 critical care. Thus, this study aims to assess the patterns of COVID-19 severity and mortality in seven countries, and to determine the risk factors of COVID-19 severity and mortality.

## Methods

### Study Design and Data Sources

This multicountry study was based on a retrospective review of medical records of hospitalized patients confirmed to have COVID-19. This study included data from Iraq, Pakistan, Sudan, Somalia, Morocco, Egypt, and Yemen. All data were extracted from hospital records (paper files) by trained data collectors who used a standardized Kobo collect form for data entry. In all participating countries, the data collected were for patients admitted to hospitals by October 2020.

In Iraq, data were collected for patients with COVID-19 who were admitted during the period between June 1 and 30, 2020, to any one of the six selected hospitals in Baghdad and one hospital in Babylon. The seven hospitals were selected out of the 10 hospitals that received patients with COVID-19 in Baghdad and Babylon because of the presence of Field Epidemiology Training Program residents who collected the data. In Sudan, the study was limited to Khartoum and Gazira states, as they were the high spots for COVID-19 in the country. The data were collected from the two main secondary isolation centers and two primary isolation centers (all hospital records of patients with a confirmed diagnosis between March 2020 and October 2020 were included). In Morocco, data were collected from the Cheikh Zaid International University Hospital of Rabat City, which was dedicated to the hospitalization of patients with COVID-19. In Egypt, data from El-Agoza Hospital in Giza governorate (a high-risk governorate) were collected between May and June 2020. El-Agoza Hospital was designated for the screening and isolation of patients with COVID-19. In Yemen, data were collected on patients with COVID-19 who were admitted to three main isolation hospitals in Sana’a City. In Pakistan, data were collected from two hospitals in Rawalpindi (Benazir Bhutto Hospital and Holy Family Hospital) and two other hospitals in Islamabad (Pakistan Institute of Medical Sciences and Pakistan Air force Hospital) of cases admitted between February and August. All four hospitals were COVID-19 treatment centers. In Somalia, data of patients admitted between March and August to De-Martino Hospital (the only COVID-19 treatment center in Mogadishu) were collected.

### Data Abstraction Form

A standardized data collection tool for all countries was developed and converted to the Kobo Toolbox form. The form consisted of four sections. The first section included patients’ baseline and demographic characteristics (age, gender, smoking history, health care worker [HCW] or not, travel history, and history of contact with patients with confirmed COVID-19). The second section included variables related to clinical history and presentation severity, signs and symptoms, and comorbidities. The third section included severity classification. Section four included outcomes (fully recovered, discharged improved, palliative discharge/disable, and death).

### Variable Definitions

WHO guidelines were used to define the history of contact (exposure during the 2 days before and the 14 days after the onset of symptoms) and travel history (history of travel 14 days before symptom onset) [20]. The severity of the disease was classified into mild (upper respiratory disease), moderate (pneumonia but no need for oxygen), severe (pneumonia and need oxygen), and critical (needs intensive care unit admission) [21]. Clinical outcomes were defined as follows: fully recovered (negative polymerase chain reaction [PCR] test before discharge or full resolution of symptoms as noted by the attending physician), discharged improved (no PCR was done before discharge but the patient was discharged based on improvement in the clinical picture), palliative discharge/disable (discharged with long-term disability due to COVID-19), and death. During the analysis, we grouped the first three categories under “survivors” and compared them to the fourth category “death.” Critical outcomes included death, palliative discharge, or disability.

### Ethical Considerations

Ethical approval was obtained from the institutional review boards in selected countries, and hospital permissions were sought to access patients’ records. Data were coded to maintain confidentiality. All data files were encrypted and saved in a secure database with limited access to the study team.

### Data Management and Analysis

Data were entered and managed using the Kobo Toolbox (a tool developed by the Harvard Humanitarian Initiative to be used for field data collection in challenging environments) and then exported to SPSS version 23 (IBM Corp) for analysis. Data were described using percentages and counts. The differences between percentages were tested using the chi-square test. Two separate multiple logistic regression analyses were conducted to determine factors associated with COVID-19 severity and mortality. The final logistic regression models included significant variables only. A *P* value less than .05 was considered statistically significant.

## Results

### Demographic and Relevant Characteristics

A total of 4141 patients were included in this study from seven countries, of which Iraq and Pakistan composed almost 60% of the sample followed by Sudan (n=1011, 24.4%). Almost 38% (n=1571) of patients aged 40-59 years, and male patients constituted 63.8% (n=2641) of the sample. About 14.6% of patients were ever smokers. Almost 4% were HCWs. A history of contact with a patient with COVID-19 was indicated by 40.8% (n=1690) of patients. Table 1 shows the demographic and relevant characteristics of patients.

**Table 1:** Demographic and relevant background characteristics of patients with COVID-19 (N=4141).

Variables		Patients, n (%)
**Country**		
	Iraq	1438 (34.7)
	Pakistan	1199 (29.0)
	Sudan	1011 (24.4)
	Somalia	230 (5.6)
	Morocco	123 (3.0)
	Egypt	71 (1.7)
	Yemen	69 (1.7)
**Age (years)**		
	<40	1193 (28.8)
	40-59	1571 (37.9)
	≥60	1377 (33.3)
**Sex**		
	Male	2641 (63.8)
	Female	1500 (36.2)
**Profession**		
	Non–health care workers	3994 (96.5)
	Health care workers	147 (3.5)
History of contact with patient with COVID-19 (yes)		1690 (40.8)
**Smoking**		
	Never	3537 (85.4)
	Ever	604 (14.6)

### Comorbidities and Clinical Manifestations

The most common symptoms on admission were fever (n=3198, 77.2%), followed by cough (n=3009, 72.7%) and shortness of breath (n=2224, 53.7%). Other common clusters encompassing musculoskeletal symptoms (myalgia or arthralgia, or backache or fatigue) were reported by 37.6% (n=1557) of patients. A cluster of enteric symptoms was less common in this study (n=398, 9.6%). Comorbidities were reported by nearly half of patients, with hypertension being the most common comorbidity (n=1021, 24.7%), followed by diabetes (n=939, 22.7%). Multiple comorbidities were also reported in almost half of the patients (n=2153, 52%; Table 2).

**Table 2:** Distribution of study participants by clinical manifestations and comorbidities (N=4141).

Variables		Patients, n (%)
**Comorbidities**		
	No comorbidity	2408 (48.2)
	Hypertension	1021 (24.7)
	Diabetes mellitus	939 (22.7)
	Heart diseases	303 (7.3)
	Renal disease	140 (3.4)
	Asthma	111 (2.7)
	Respiratory diseases (COPD^a^ and other respiratory diseases)	61 (1.5)
	Malignancy	62 (1.5)
	Immune compromising conditions	45 (1.1)
	Liver disease	22 (0.5)
**Symptoms**		
	Asymptomatic	124 (3.0)
	Fever	3198 (77.2)
	Cough	3009 (72.7)
	Shortness of breath	2224 (53.7)
	Musculoskeletal manifestations	1557 (37.6)
	Sore throat	683 (16.5)
	Headache	664 (16.0)
	Loss of taste or smell	474 (11.4)
	Chest pain	458 (11.1)
	Gastrointestinal or gastroenteric symptoms (nausea/vomiting/diarrhea/abdominal pain)	364 (8.8)
	Sputum production	156 (3.8)
	Nasal congestion	118 (2.8)
	Conjunctival congestion	37 (0.9)
	Hemoptysis	36 (0.9)
	Other symptoms	134 (3.2)

^a^COPD: chronic obstructive pulmonary disease.

### Factors Associated With COVID- 19 Severity and Mortality

Of all patients, 27.6% (n=1143) had mild disease, 22.7% (n=940) had moderate disease, 28.9% (n=1197) had severe disease, and 20.7% (n=857) had critical disease. The bivariate analysis had shown that increased age, non–health professions, ever smoking, and comorbidity history, particularly hypertension, diabetes mellitus, heart diseases, and respiratory diseases, were significantly associated with severe or critical COVID-19. Whereas mortality was consistently associated with the aforementioned factors in addition to the male sex, cerebrovascular disease, and malignancy (Table 3).

**Table 3:** Bivariate analysis of factors associated with COVID-19 severity and mortality.

Variable		Severity					Mortality				
		Mild-moderate, n (%)	Severe-critical, n (%)	*P* value		Survivors, n (%)		Deaths, n (%)		*P* value	
**Sex**					.45						.005
	Male	1318 (49.9)	1323 (50.1)			2132 (80.7)		509 (19.3)			
	Female	767 (51.1)	733 (48.9)			1263 (84.2)		237 (15.8)			
**Age (years)**					<.001						<.001
	<40	849 (71.2)	344 (28.8)			1115 (93.5)		78 (6.5)			
	40-59	779 (49.6)	792 (50.4)			1334 (84.9)		237 (15.1)			
	≥60	457 (33.2)	920 (66.8)			946 (68.7)		431 (31.3)			
**Working in health care setting**					<.001						<.001
	Non–health care workers	1965 (49.2)	2029 (50.8)			3254 (81.5)		740 (18.5)			
	Health care workers	120 (81.6)	27 (18.4)			141 (95.9)		6 (4.1)			
Positive history of contact with patient with COVID-19		1116 (66.0)	574 (34.0)	<.001		1456 (86.2)		234 (13.8)		<.001	
**Smoking**					<.001						.29
	Ever	228 (37.7)	376 (62.3)			486 (80.5)		118 (19.5)			
	Never	1857 (52.5)	1680 (47.5)			2909 (82.2)		628 (17.8)			
**Comorbidities**		592 (34.2)	1141 (65.8)	<.001		1237 (71.4)		496 (28.6)		<.001	
	Hypertension	353 (34.6)	668 (65.4)	<.001		714 (69.9)		307 (30.1)		<.001	
	Diabetes mellitus	319 (34.0)	620 (66.0)	<.001		650 (69.2)		289 (30.8)		<.001	
	Heart diseases	91 (30.0)	212 (70.0)	<.001		190 (62.7)		113 (37.3)		<.001	
	Renal disease	25 (17.9)	115 (82.1)	<.001		108 (77.1)		32 (22.9)		.13	
	Asthma	50 (45.0)	61 (55.0)	.26		91 (82.0)		20 (18.0)		>.99	
	Respiratory diseases	21 (34.4)	40 (65.6)	.01		38 (62.3)		23 (37.7)		<.001	
	Malignancy	21 (33.9)	41 (66.1)	.009		43 (69.4)		19 (30.6)		.009	
	Immunocompromising conditions	12 (26.7)	33 (73.3)	.001		39 (86.7)		6 (13.3)		.41	
	Liver disease	8 (36.4)	14 (63.6)	.19		16 (72.7)		6 (27.3)		.26	
	Tuberculosis	19 (33.9)	37 (66.1)	.01		42 (75.0)		14 (25.0)		.17	
	Cerebrovascular disease	8 (42.1)	11 (57.9)	.47		12 (63.2)		7 (36.8)		.03	

The multiple logistic regression indicated that increased age and smoking were significantly associated with severity (*P*<.001). Patients older than 60 years were three times more likely to develop a severe or critical form of COVID-19 compared to patients younger than 40 years. Patients with smoking history were 10 times more likely to develop severe or critical disease course compared to nonsmokers (odds ratio [OR] 9.7, 95% CI 5.1-18.5; *P*<.001). Not having a history of contact with patients with COVID-19 (OR 2.8, 95% CI 2.4-3.2; *P*<.001) and being non-HCWs (OR 2, 95% CI 1.2-3.1; *P*=.004) were also significantly associated with developing a severe form of COVID-19. Of comorbidities, renal diseases (OR 3.3, 95% CI 2.1-5.2; *P*<.001), heart diseases (OR 1.7, 95% CI 1.3-2.2; *P*<.001), diabetes (OR 1.4, 95% CI 1.2-1.7; *P*<.001), and hypertension (OR 1.2, 95% CI 1-1.5; *P*=.03) were associated with increased odds of severe disease (Table 4).

**Table 4:** Multiple logistic regression of factors associated with COVID-19 severity and mortality.

Covariates		Factors associated with COVID-19 severity			Factors associated with COVID-19 mortality	
Sex (female vs male)		—^b^	N/A^c^	0.8 (0.7-1)		.02
**Age (years)**						
	<40 (reference)	N/A	N/A	N/A		N/A
	40-59	2 (1.7-2.4)	<.001	1.9 (1.5-2.5)		<.001
	≥60	3.1 (2.6-3.8)	<.001	4 (3.1-5.3)		<.001
**Profession**						
	Health care workers (reference)	N/A	N/A	N/A		N/A
	Non–health care workers	2 (1.2-3.1)	.004	2.4 (1-5.6)		.04
History of contact with patient with COVID-19 (no vs yes)		2.8 (2.4-3.2)	<.001	1.4 (1.2-1.7)		<.001
Diabetes mellitus (yes vs no)		1.4 (1.2-1.7)	<.001	1.6 (1.3-1.9)		<.001
Hypertension (yes vs no)		1.2 (1-1.5)	.03	1.3 (1.1-1.6)		.003
Heart diseases (yes vs no)		1.7 (1.3-2.2)	<.001	1.8 (1.4-2.3)		<.001
Renal disease (yes vs no)		3.3 (2.1-5.2)	<.001	—		N/A
Respiratory diseases (yes vs no)		—	N/A	2.1 (1.2-3.7)		.009
Malignancy (yes vs no)		—	N/A	1.8 (1-3.3)		.04
Symptomatic (yes vs no)		—	N/A	4.5 (1.6-12.6)		.004
Smoking (ever vs never)		9.7 (5.1-18.5)	<.001	—		N/A

^a^OR: odds ratio.

^b^Not entered in the model because the variable was not statistically significant.

^c^N/A: not applicable.

Increasing age was a strong predictor of mortality in hospitals, as the older age group (>60 years) was associated with an increased odds of death by almost four times compared to the younger age category (OR 4, 95% CI 3.1-5.3; *P*<.001). The history of contact with patients with COVID-19s and being a non-HCW had higher odds of mortality. Female sex was associated with lower odds of mortality (OR 0.8, 95% CI 0.7-1.0; *P*=.02). Comorbidities associated with high odds of morality included respiratory diseases (OR 2.1, 95% CI 1.2-3.7; *P*=.009), heart diseases (OR 1.8, 95% CI 1.4-2.3; *P*<.001), malignancy (OR 1.8, 95% CI 1.0-3.3; *P*=.04), and diabetes mellitus (OR 1.6, 95% CI 1.3-1.9; *P*<.001). Renal diseases and smoking history had no significant association with mortality (Table 4).

## Discussion

### Principal Results

The data of a cohort of patients with COVID-19 hospitalized in seven different countries were collected and analyzed. The proportion of hospitalized patients with severe or critical illness in this study (49.6%) is remarkably higher than what is being reported from other parts of the world on hospitalized patients with COVID-19 [22-25]. To our knowledge, only one study from Wuhan reported a severity profile as high as 49.1% [26]. However, given that the study was conducted among hospitalized patients rather than in the community, these proportions are not a true representative of the COVID-19 severity spectrum in this region. At the beginning of the pandemic, the health care facilities used to admit patients with COVID-19 of different severity for the sake of isolation and treatment of those patients. Later, with the increase in the number of patients, the health care facilities in most of these countries started to reserve the already limited hospital beds to treat the more severe cases, which in turn shifted the majority of mild/moderate cases to be treated at home.

We found older age, diabetes mellitus, hypertension, and heart diseases to be associated with both severity and mortality. Furthermore, ever smoking and renal diseases were associated with severity but not mortality, while male gender, respiratory diseases (COPD or other respiratory diseases), and malignancy were associated with mortality but not severity. The latter category is of special importance, given the fact that these patients might die even if they do not experience severe or critical illness. The majority of these findings are consistent with previous studies on COVID-19 severity or mortality [27-31].

### What Is Already Known on This Topic

Unraveling the pathophysiology of COVID-19 is still a work in progress. Nevertheless, the role of angiotensin-converting enzyme 2 (ACE2) receptors is widely recognized as a key player in the disease process. ACE2 is a metallopeptidase that is expressed in various human organs [32] and is thought to be the cell entry point for SARS-CoV-2 [33]. Among others, it has been found on cell membranes of the nasal epithelium, alveolar epithelial cells of the lungs, the small intestine enterocytes, vascular endothelial cells, and cardiovascular system cells [32-35]. It is believed that negative regulation of the renin-angiotensin system (RAS) is a main function of ACE2. RAS has been linked to lung injury [36,37], which has led to concluding that ACE2 has a protective effect on the lungs [38,39] and that its pre-existing deficiency might lead to a more severe or even fatal COVID-19 [40].

Older patients, males, and patients with type 2 diabetes mellitus were frequently found to have severe or fatal COVID-19 in epidemiological studies. Interestingly, they were also found to have lower ACE2 expression, which might help explain why they are disproportionately affected by poor COVID-19 outcomes [41-43]. Furthermore, age (the earliest and most widely recognized predictor of COVID-19 severity and mortality) showed the strongest correlation with low ACE2 expression [41].

In addition to ACE2 downregulation, other biological and nonbiological factors might help explain some of our findings. For example, differences in the biological and molecular level in older patients (as compared to younger ones) might have their contribution to the disease course [44]. In particular, two cardinal features of the aging immune system are probably the culprits: immune senescence (a general decline in the overall performance of the immune system, innate and acquired) and inflamm-aging (a systemwide persistent proinflammatory status) [44-46]. However, some have argued that age-associated comorbidities, rather than age itself, are the major factor behind these findings [47]. However, in this paper, we found that age is an independent risk factor on top of the comorbidities from the multiple logistic regression (Table 4).

Diabetes mellitus is another risk factor where the dysregulated immune response is thought to play a role in a patient’s susceptibility to critical COVID-19 outcomes. Similar to aging, patients with diabetes are in a state of low-grade chronic inflammation [48]. Furthermore, diabetes is associated with reduced activity of natural killer cells and impaired cell-mediated adaptive immunity (chemotaxis, phagocytosis, cytokine secretion, and T-cell abnormalities) [48].

The fact that an overactive RAS is a key aspect of the pathogenesis of CVD (heart diseases and hypertension) [49,50] might explain the association between CVD and fatal COVID-19. An already existing abnormal cytokine profile in patients with these comorbidities can be part of the explanation as well [51-53]. Knowing that cytokine storm is frequently linked to severe and fatal COVID-19 strengthens the biological plausibility of this explanation [8,54].

Similar to CVD, some renal diseases are characterized by an overactive RAS [55] and linked to dysregulated cytokine function and proinflammatory status [56,57]. Although patients with renal diseases were more likely to have severe illness in our study, we found no association with mortality. This is inconsistent with previous reports [58,59], which might be explained by differences in renal disease definitions and severity levels of the studied patients. These same reasons might explain why, unlike other studies [60], we found no association between liver disease and critical COVID-19 outcomes.

The defective local physiological function of the respiratory system in patients with COPD and other respiratory diseases could explain their predisposition to worse COVID-19 outcomes. Conversely, asthma was not associated with COVID-19 severity or mortality. The evidence in the literature on asthma has been inconclusive [61].

### Comparison With Prior Work

Despite the fact that smoking is probably underestimated in our study due to associated stigma, particularly among women, we were able to find an association with severity. However, this same underestimation might be the reason why, unlike previous reports [17], we could not find an association with mortality. Another reason is the fact that in our study, due to small cell size, we combined former and current smokers under the same category, while in other studies, mortality was found to be associated with current rather than former smokers [17].

Unexpectedly, we also found not working in a health care setting and not having a history of contact with a known COVID-19 case to be associated with both severity and mortality. These findings should be interpreted with caution though; patients with a known COVID-19 contact history might be better informed and more likely to seek help early and thus less likely to have critical outcomes.

There is also the possibility that the history of contact is underreported mainly for two reasons. First, the stigma around the disease may prevent many people from telling everyone around them; thus, many people may not realize that they had been in contact with a patient with COVID-19.

Second, the societies in the EMR are among the most social in the world and being friendly to strangers is the social norm. In Iraq, for example, religious mass gatherings where strangers often socialize without observing social distancing is a frequent occurrence. There is a decent possibility that some of the patients in this study might, unknowingly, had come in contact with presymptomatic or mild COVID-19 cases during one of these events.

Similar to our study, a meta-analysis by the American Journal of Emergency Medicine [62] found that HCWs were less likely to have severe or critical disease, or to die of COVID-19. The proposed explanation by the authors seems to hold for our study as well: that HCWs tend to come from a younger age demographic and have fewer comorbidities [62,63]. Further analysis of the characteristics of HCWs in our study revealed that only 4.8% of them were 60 years or older, while the majority (89/147, 60.5%) were younger than 40 years, and 34.7% (51/147) were aged between 40 and 59 years. Additionally, only 4.1% (6/147) had comorbidities.

### What This Study Adds

Almost a year has passed since the COVID-19 pandemic started, and still the best intervention we have to fight it with is nonpharmaceutical measures. There is no definite treatment to date, and although good news on effective vaccines is emerging, it might take a few years until enough of the population has access to a vaccine. That is why, in the hospital setting, it is still imperative to identify which patients are at higher risk of developing severe disease or dying of COVID-19. Although many studies reported on this worldwide, to our best knowledge, this is the first large multicenter study coming out of the EMR.

### Limitations

This study has its limitations. First, the study was conducted in a hospital setting, which limits our ability to generalize our findings to the general population. Second, given that in many hospitals in the selected countries hospital admissions were reserved for the most severe cases, our study might be biased toward more severe outcomes. Finally, some variables might be underreported due to the inability of the HCWs in the overwhelmed facilities to collect all data for all patients.

### Conclusions

This study reports on risk factors of COVID-19 severity and mortality in the seven countries. In a hospital setting, health care providers should be more attentive to older patients, men, smokers, and patients with certain comorbidities (diabetes, hypertension, heart diseases, COPD, malignancy, and renal diseases), as they were shown to be more likely to have severe or fatal COVID-19 in our study.

## Abbreviations

ACE2: angiotensin-converting enzyme 2
CFR: case-fatality rate
COPD: chronic obstructive pulmonary disease
CVD: cardiovascular disease
EMR: Eastern Mediterranean Region
HCW: health care worker
OR: odds ratio
PCR: polymerase chain reaction
RAS: renin-angiotensin system
WHO: World Health Organization
